# The Anterior BNST Is Required for Novelty-Driven Social Interaction

**DOI:** 10.1523/JNEUROSCI.1743-25.2026

**Published:** 2026-01-21

**Authors:** Jessica T. Jacobs, Mikaela L. Aholt, Taylor Lineberry, Magdalene P. Adjei, Elana Qasem, Sophia Aaflaq, Sandria W. Athul, Buffy S. Ellsworth, Jacob C. Nordman

**Affiliations:** Division of Molecular and Integrative Physiology, Department of Biomedical Sciences, Southern Illinois University School of Medicine, Carbondale, Illinois 62903

**Keywords:** bed nucleus of the stria terminalis, familiarity, social behavior, social cognition, social novelty

## Abstract

Social novelty preference—the tendency to interact more with unfamiliar than familiar conspecifics—is conserved across species and disrupted in disorders such as autism spectrum disorder, schizophrenia, and social anxiety. While the hippocampus and related circuits are known to encode social recognition memory, the mechanisms that translate familiarity signals into behavioral differences remain unclear. Here, we show that male mice exhibit a robust preference for engaging with unfamiliar over familiar conspecifics. Using c-Fos labeling, RNAscope, immunohistochemistry, and fiber photometry, we found that inhibitory and DRD1-expressing neurons in the dorsal subdivision of the anterior bed nucleus of the stria terminalis (BNSTa) are broadly activated during social and novel–object interaction. However, chemogenetic inhibition of the BNSTa selectively suppressed interaction with unfamiliar conspecifics while leaving familiar and novel–object interactions unaffected. These findings identify the BNSTa as a critical node that promotes novelty-driven social engagement, revealing a circuit mechanism for social novelty preference. Because deficits in novelty processing are central to multiple neuropsychiatric disorders, our results highlight the BNST as a potential locus of dysfunction linking social recognition to behavior.

## Significance Statement

Recognizing whether a social partner is familiar or unfamiliar is fundamental for survival, yet most research has overlooked how familiarity shapes social behavior. Here we identify the anterior bed nucleus of the stria terminalis (BNSTa) as a critical regulator of interactions with unfamiliar conspecifics. Although BNSTa inhibitory neurons are broadly engaged during social encounters, chemogenetic inhibition selectively suppresses social engagement with strangers while leaving interactions with familiar conspecifics and objects intact. These findings reveal a previously unexplored role of the BNSTa in promoting novelty-driven social interaction. Because disruptions in social novelty processing are a hallmark of conditions such as autism spectrum disorder, schizophrenia, and social anxiety, our results provide insight into how BNST dysfunction may contribute to psychiatric social deficits.

## Introduction

Social behavior has been strongly selected for throughout evolution because it confers substantial survival and reproductive advantages; accordingly, the social environment is one of the most consistent predictors of health and longevity across mammalian species (Yang et al., 2016; Ellis et al., 2019; Snyder-Mackler et al., 2020). A fundamental component of social behavior is the ability to recognize conspecifics as familiar or unfamiliar. Interaction with a novel individual carries both potential risk and reward, and so determining conspecific identity is critical for survival. In turn, many social species exhibit a social novelty preference, spending more time with unfamiliar individuals (Thor and Holloway, 1982; Gothard et al., 2004; Moy et al., 2004; Smith et al., 2015; do Nascimento and Maximino, 2023). Deficits in novelty processing are prominent in neuropsychiatric conditions characterized by disrupted social functioning, including autism spectrum disorder, schizophrenia, and anxiety disorders (Pierce et al., 2004; Sachs et al., 2004; Blackford et al., 2011; Williams et al., 2013). Understanding how the brain governs responses to familiar versus unfamiliar conspecifics is therefore essential for explaining both adaptive social behavior and its disruption in psychiatric disease.

The bed nucleus of the stria terminalis (BNST) is a promising candidate region for guiding social behavior based on familiarity. Although best known for its role in anxiety and sustained fear (Walker et al., 2003; Walker and Davis, 2008; Davis et al., 2010; Alvarez et al., 2011), the BNST is also a core node of the evolutionarily conserved social behavior network (Newman, 1999; O’Connell and Hofmann, 2011). Early studies demonstrated that BNST vasopressin neurons regulate male-typical social behaviors, including scent marking and aggression (De Vries et al., 1981; Bester-Meredith et al., 1999; Albers, 2012), and subsequent work has implicated BNST circuits in mating, parental care, affiliative behavior, and social anxiety (Kim et al., 2015; Perkins et al., 2017; Duque-Wilckens et al., 2018; Klampfl and Bosch, 2019; Flanigan and Kash, 2020). Additional findings suggest the role of the BNST in novelty- or familiarity-dependent social processes. Disrupting vasopressin or oxytocin signaling in the BNST impairs social recognition, and chemogenetic manipulation of the BNST can restore social novelty preference following early-life stress (Dumais et al., 2016; Whylings et al., 2020; Emmons et al., 2021). Together, these studies suggest that BNST circuits bias social responses in novelty-related contexts, though whether BNST activity directly regulates behavioral differences between familiar and unfamiliar conspecifics remains unknown.

Recent work by Jacobs and colleagues suggests that BNST inactivation biases macaques toward a behavioral profile characteristic of familiar social interaction (Jacobs et al., 2023). Pharmacological inactivation of the anterior BNST (BNSTa)—a region implicated in affiliative and motivated behaviors (Newman, 1999; Dong and Swanson, 2006; Fox et al., 2015; Lebow and Chen, 2016; Flanigan and Kash, 2020; Jacobs et al., 2023)—increased total social contact without altering active engagement. Classic rodent studies show that familiar pairs spend less time in social investigation and more time in passive contact compared with unfamiliar pairs (Kareem and Barnard, 1982; Thor and Holloway, 1982; Kareem, 1983; Moy et al., 2004), closely resembling the behavioral profile observed following BNST inactivation. Based on these converging findings, we hypothesized that BNSTa neurons are necessary for enhanced engagement with unfamiliar conspecifics.

To test this hypothesis, we combined immediate-early gene labeling, cell-type identification, real-time calcium imaging, and causal manipulations of the BNSTa in familiar and unfamiliar mice during or after engaging in a free social interaction test. We further analyzed dorsal and ventral BNSTa subdivisions separately, as prior work suggests functional specialization along this axis, with dorsal regions linked to approach-related behaviors and ventral regions associated with social threat processing (Fox et al., 2015; Lebow and Chen, 2016; Flanigan and Kash, 2020). Together, this approach allowed us to directly test how BNSTa circuits regulate behavioral differences based on social familiarity, providing unprecedented insight into how BNST dysfunction may contribute to psychiatric social deficits.

## Materials and Methods

### Animals

All animal protocols were approved by the Animal Care and Use Committee of Southern Illinois University School of Medicine. All male CD1 mice used in this study were purchased from Charles River Laboratories, culled prior to arrival in our animal facility, and then group-housed with their litter mates in a 19.37 × 18.06 × 39.83 cm polycarbonate cage (Allentown) on a reverse 12 h light cycle (lights off 8:30 A.M. to 8:30 P.M.) with *ad libitum* access to water and food. We refer to the group-housed mice as familiar if they had persistent and unrestricted tactile and olfactory contact from birth (indicated by Charles River Laboratories). Unfamiliar animals were characterized as having no previous interaction prior to testing (Adjei et al., 2025). Surgical procedures, outlined below, were performed at 5–6 weeks of age and then the mice were group-housed for an additional 3–4 weeks. All behavioral testing was conducted at the same age of 8–9 weeks.

### Behavior experiments

#### Free social interaction test

Group-housed 8-to-9-week-old mice were transferred to a darkened behavior room and left to acclimate for at least 1 h prior to testing. Familiar or unfamiliar CD1 mice were then placed in a novel, high-walled cage and allowed to freely interact for 5 min. We elected to use a novel cage to reduce the effect of territoriality on social behavior (Koolhaas et al., 2013). Animal behavior was captured via an overhead tripod-mounted video camera. Behavior videos were reviewed and manually scored using BehaviorCloud by a researcher blind to the experimental conditions. A subset of behavioral videos was scored by two independent raters, with high inter-rater reliability (ICC > 0.95). Social behavior is defined as anogenital sniffing, nonaggressive investigation, and flank rubbing (Blanchard and Blanchard, 1977; Koolhaas et al., 2013; Nordman and Li, 2020), and social behavior was quantified as the total interaction time, the number of interactions, the average duration of interaction, and latency to the first interaction (J. C. Nordman et al., 2020, 2022; J. Nordman et al., 2020; Bartsch et al., 2023a; Mojahed et al., 2024).

#### Object interaction test

Postsurgical group-housed 8-to-9-week-old mice were transferred to a darkened behavior room and left to acclimate for at least 1 h prior to testing. Mice were then placed into a novel, high-walled cage and allowed to freely interact with either a familiar or unfamiliar object for 5 min. Familiar objects were defined as objects identical to those left in the group-housed cage for 1 week prior to testing. Animal behavior was captured via an overhead tripod-mounted video camera and analyzed using BehaviorCloud using either its automated analysis software or by hand by a researcher blind to the experimental conditions. Behavior was quantified as the total interaction time (within 40 mm of object) and total ambulatory time.

### Immunohistochemistry, RNAscope, and viral confirmation

Mice were transcardially perfused with 4% paraformaldehyde in phosphate-buffered saline solution (PBS). Brains were removed and postfixed at 4°C overnight and then cryoprotected overnight in 15% sucrose (in PBS) followed by 30% sucrose (in PBS). Brains were cut into 30-µm-thick sections for immunohistochemistry and viral injection confirmation and 15-µm-thick sections for RNAscope analysis using a cryostat (Leica CM3050-S). Slices were then stored in PBS as floating sections until use.

For immunohistochemistry experiments, free-floating brain sections were blocked in PBS containing 10% goat serum, 1% bovine serum albumin, and 0.03% Triton X-100 for 2 h at room temperature. Sections were then incubated overnight at 4°C with primary antibodies directed against c-Fos (rabbit, 1:2000, Abcam, ab190289 or mouse, 1:1,000, Proteintech, 66590-1-Ig), DRD1 (rabbit, 1:200, Proteintech, 17934-1-AP), and PKCδ (mouse, 1:250, BD Biosciences, 610398), as indicated for each experiment. Following primary incubation, sections were washed and incubated for 1 h at room temperature with species-appropriate Alexa Fluor-conjugated secondary antibodies (1:200; Thermo Fisher Scientific). Sections were then mounted onto slides with Vectashield HardSet Antifade Mounting Medium containing DAPI (Vector Laboratories).

For RNAscope experiments, we the labeled BNSTa tissue with probes to detect vGAT [vGAT (SLC32A1), Channel 2 (Alexa Fluor 488), catalog #319191-C2, Accession Number NM 009508.2, Position 894-2037] and c-Fos [FOS (D12Rfj1), Channel 3 (Alexa Fluor 647), catalog #316928-C3, Accession Number NM 010234.2, Position 407-1427). We used an Olympus FV1000 laser point-scanning confocal microscope to collect 63× 1 μm *z*-stack images. Images where then analyzed for total expression and colocalization of vGAT and c-Fos using Fiji.

To confirm the location of viral injection, sections were imaged with an EVOS fluorescent microscope using a 10× (NA 0.45) objective or a Nikon fluorescent microscope (ECLIPSE Ti2-E) using a 10× (WD 20) objective.

### Surgical procedures

All procedures were performed on 5-to-6-week-old CD1 male mice that were anesthetized with isoflurane (3% for induction and 1–2% for maintenance) and then placed onto a stereotaxic frame (David Kopf Instruments) for viral injection and fiber implantation.

For fiber photometry experiments, we unilaterally injected 250 nl of AAV9-hSyn-GCaMP7f.WPRE SV40 (GCaMP7f, Addgene #104488, 2.3 × 10^13^ vg/ml) or pAAV9-hSyn-EGFP (GFP, Addgene #202190, 1.9 × 10^13^ vg/ml) into the BNSTa (AP, 0 mm; ML, +0.75 mm; DV, −3.85 mm) of 5-to-6-week-old CD1 male mice using a 5 μl gas-tight Neuros Hamilton Syringe coupled to a 33 gauge stainless steel needle ejected at a rate of 30 nl/min by a syringe pump (World Precision Instruments). In a subset of animals, injections and fiber implants were made in the contralateral hemisphere to assess the potential influence of lateralization on BNSTa responses. After injection, the syringe was left in place for an additional 10 min and then slowly withdrawn. After viral injection, optical fiber ferrules (photometry, diameter 1.25 mm and depth 3.7 mm, black; RWD Life Science) were implanted into the dorsal BNSTa. The fibers were secured to the skull with stainless steel screws (1.6 mm, Protech International), a thin layer of Metabond (Parkell) and then a larger layer of acrylic dental cement (Lang Dental). Once the cement had fully cured, animals were placed back in their home cage on a preheated pad at 37°C. Ketoprofen or meloxicam were administered for 3 d postsurgery. Mice were allowed to recover for 3–4 weeks before free social or object interaction testing.

For chemogenetic experiments, a bilateral craniotomy was made, and 400 nl of pAAV9-hSyn-hM4D(Gi)-mCherry (hM4Di, Addgene #50475, 2.6 × 10^13^ vg/ml) or pAAV9-hSyn-mCherry (mCherry, Addgene #114472, 2.1 × 10^13^ vg/ml) was injected into each BNSTa (AP, 0 mm; ML, ±0.75 mm; DV, −3.85 mm) using the method described above. Again, ketoprofen (5 mg/kg) was administered for 3 d postsurgery. Mice were allowed to recover for 3–4 weeks before the free social or object interaction test.

### Fiber photometry recordings

On test day, surgical animals were transferred from their housing room to a darkened behavior room and left to acclimate for 1 h prior to recording. Calcium activity was recorded using a tricolor fiber-photometry rig (R820, RWD Life Science). Although the system houses 410, 470, and 560 nm LEDs, only the 410 nm (isosbestic reference) and 470 nm (GCaMP7f excitation) channels were employed. Both LEDs were routed through a dual-band 410/470 nm dichroic and a 200 µm core, 0.37 NA patch cord to a chronically implanted ferrule (RWD Life Science) positioned into the dorsal BNSTa. Excitation power at the ferrule tip was set to 20 µW (Thorlabs photodiode meter) to minimize bleaching while maintaining signal to noise. Fluorescence returned through the same fiber and was captured by the system's integrated sCMOS detector at 60 Hz; synchronized behavioral video was acquired simultaneously at 30 fps (640 × 480 px).

Motion artifacts and bleaching were corrected by fitting the 410 nm trace to the 470 nm trace with least squares regression and computing Δ*F*/*F* = (470 nm − fitted 410 nm) / fitted 410 nm. The resulting trace was *z*-scored to a 2 s pre-event baseline for each bout (social interaction, object interaction, or general locomotion). Area under the curve (AUC) values for the 2 s postevent window were compared with baseline AUCs using nested *t* tests, with bouts nested within animals; bouts containing other events within the baseline window were omitted automatically by custom MATLAB scripts. All animals showed on-target viral expression and fiber placement, resulting in the inclusion of all recordings.

### Chemogenetic experiments

On the test day, surgical animals were transferred from their housing room to a darkened behavior room and left to acclimate for 1 h prior to testing. hM4Di- or mCherry-expressing mice were injected with 0.9% saline (vehicle) or 2 mg/kg clozapine *N*-oxide (CNO; Sigma-Aldrich), the effective dose in our previous studies (Bartsch et al., 2023b; Mojahed et al., 2024; Adjei et al., 2025), 30 min before the free social or object interaction test.

### Experimental design and statistical analyses

The GraphPad Prism software was used for statistical analysis. All analyses were blind to condition. Power analyses for each experiment were calculated using G*power and adjusted based on previous studies (J. C. Nordman et al., 2020; Adjei et al., 2025). All analyses assume a standard deviation of 20%, 1-β of 0.8, and an *α* of 0.05. Assumptions were checked for all experimental models using the Shapiro–Wilk test of normality and Levene's test for equality of variance. *P* < 0.05 was considered significant, and all tests were two-tailed. For photometry analysis, nested *t* tests were chosen to preserve trial-level information while accounting for animal-level clustering; analyses using per-animal averages yielded qualitatively identical results (data not shown). All data were presented as individual data points or expressed as mean ± SEM. Details can be found in Table S1.

## Results

### Unfamiliar mice engage in social interaction more than familiar mice

**Figure 1.**
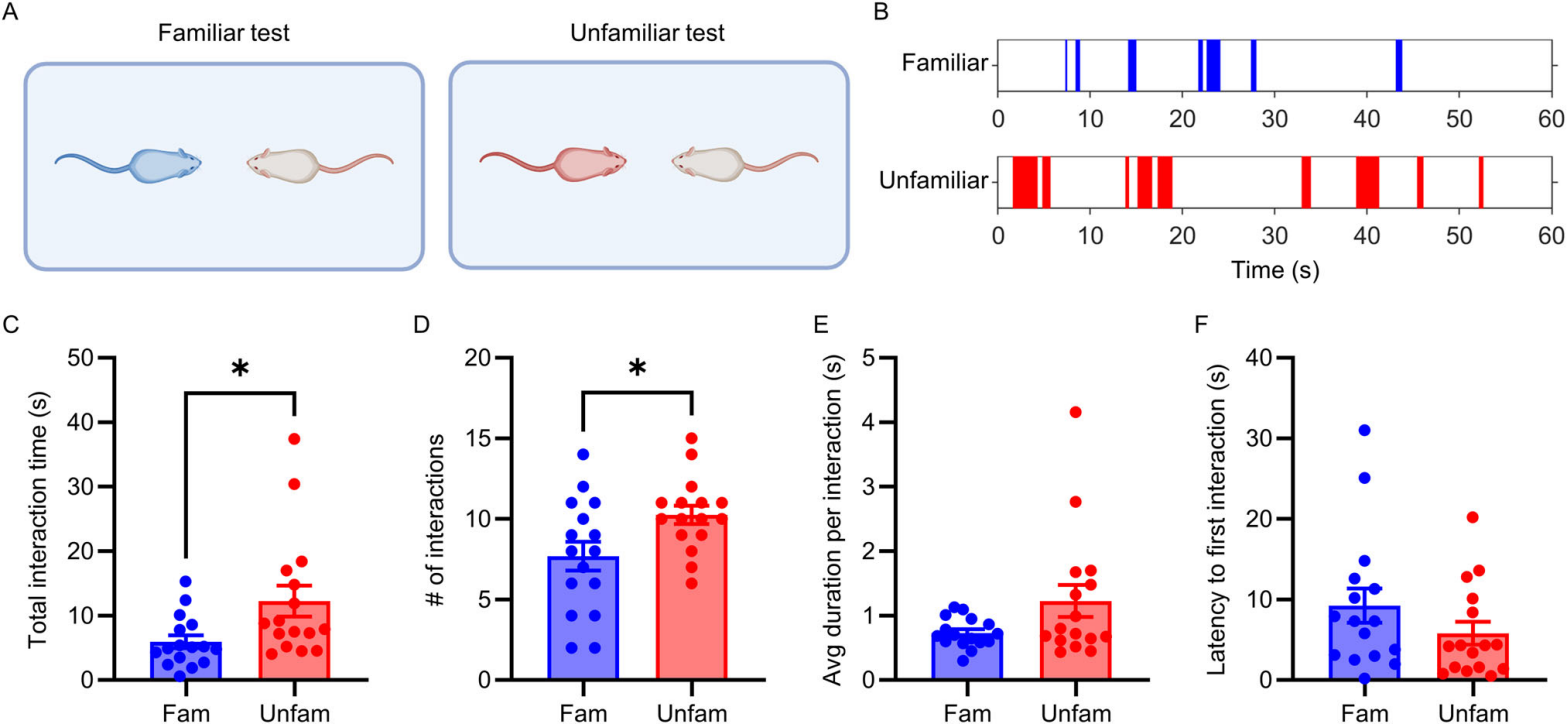
Unfamiliar mice interact longer than familiar mice. ***A***, Free social interaction test. ***B***, Representative raster plots of social interaction (SI) between familiar or unfamiliar pairs of mice. Colors refer to the identity of the mice performing the social behavior (blue is familiar; red is unfamiliar). ***C–F***, Quantification of social behavior during the free familiar (fam) or unfamiliar (unfam) social interaction test. Mean ± SEM. *p < 0.05.

We began by confirming previous reports that unfamiliar mice interact more with unfamiliar conspecifics compared with familiar conspecifics (Kareem and Barnard, 1982; Kareem, 1983; Moy et al., 2004). To test this, we used a free social interaction test in which two adult CD1 mice were placed in a novel test arena and allowed to freely interact (Fig. 1*A*). We chose CD1 mice because of their relatively high sociality (Moy et al., 2004; S. S. Lee et al., 2025). As described in the Materials and Methods section, we used a novel arena to avoid any effect of territoriality on social behavior.

Consistent with previous work, our results show that unfamiliar mice interact more than familiar mice (Fig. 1*B*), both in terms of total time (*U* = 65; *p* = 0.017; Fig. 1*C*; Table S1) and number of interactions (*t*_(25.74)_ = 2.432; *p* = 0.022; Fig. 1*D*). Average duration of interactions (*U* = 95; *p* = 0.220; Fig. 1*E*) and latency to first interaction (*U* = 95; *p* = 0.220; Fig. 1*F*) did not differ between familiar and unfamiliar pairs.

### Social interaction activates vGAT+ and DRD1+ dorsal BNSTa neurons regardless of familiarity

**Figure 2.**
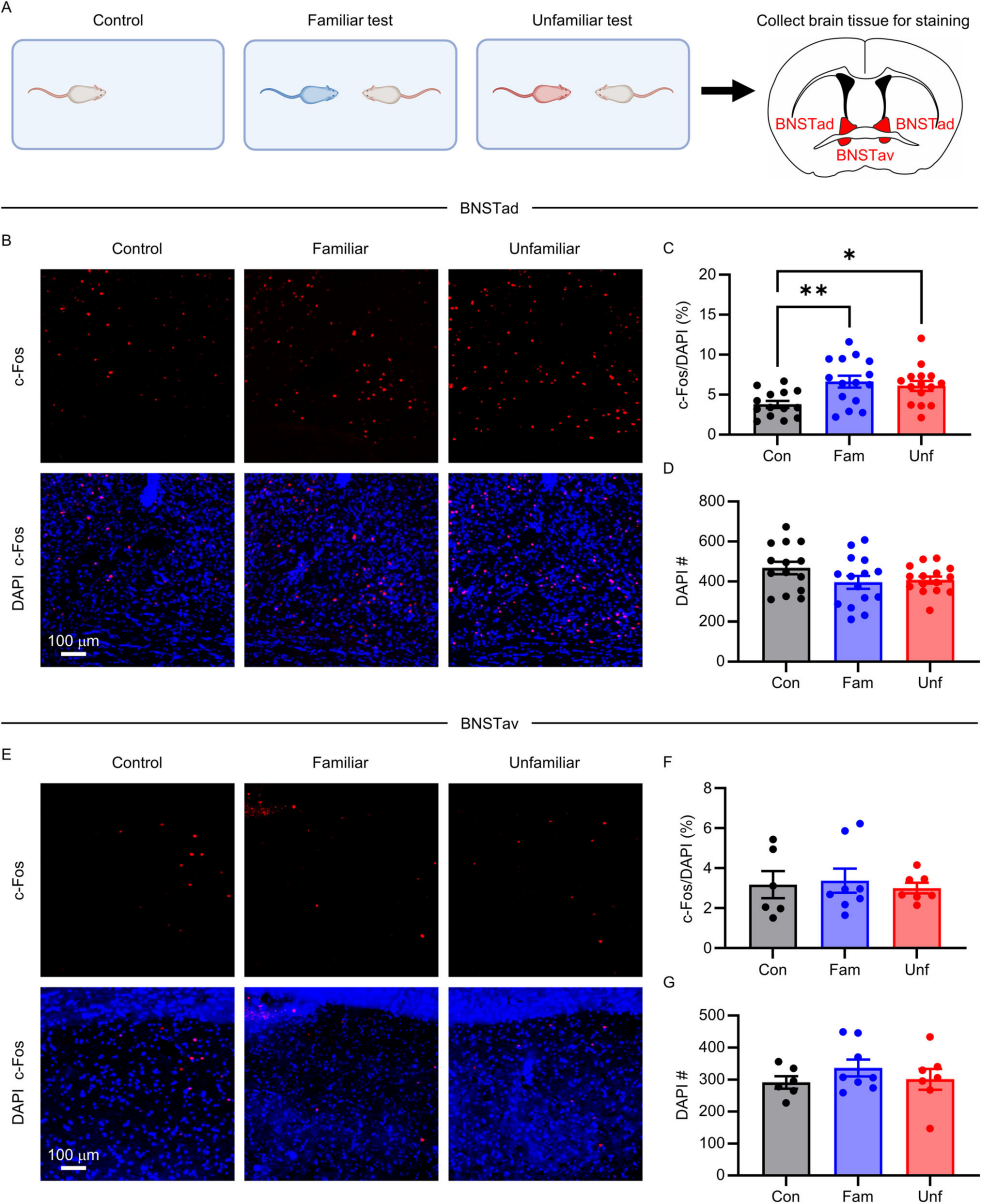
Social interaction activates the dorsal, but not ventral, BNSTa. ***A***, Experimental paradigm. ***B***, ***E***, Representative images of c-Fos+ and DAPI+ cells in the dorsal BNSTa (BNSTad; ***B***) and ventral BNSTa (BNSTav; ***E***) after the familiar or unfamiliar free social interaction test or control condition (mice left in the arena without a conspecific). Scale bar, 100 mm. ***C***, ***D***, ***F***, ***G***, The percentage of c-Fos–labeled DAPI+ cells (***C***) and total DAPI+ cells in the dorsal (***C***, ***D***) and ventral (***F***, ***G***) BNSTa for each condition. Mean ± SEM. *p < 0.05; **p < 0.01.

Having established that social interaction differs based on familiarity, we next asked whether neuronal activity within the BNSTa is differentially engaged during interaction with familiar versus unfamiliar conspecifics. To address this, we quantified expression of the immediate-early gene c-Fos, a marker of recent neuronal activation, in the dorsal and ventral subdivisions of the BNSTa following free social interaction with either a familiar or unfamiliar conspecific or after an equivalent period spent alone in the test arena (control condition; Fig. 2*A*).

Interaction with either a familiar or unfamiliar conspecific resulted in a significantly higher percentage of c-Fos+ DAPI-labeled cells in the dorsal, but not the ventral, BNSTa relative to the control condition (dorsal, *F*_(2,41)_ = 5.716; *p* = 0.0065; Fig. 2*B*,*C*; ventral, *F*_(2,18)_ = 0.1255; *p* = 0.8828; Fig. 2*E*,*F*; Table S1). The total number of DAPI-labeled cells did not differ across conditions in either subregion (dorsal, *F*_(2,41)_ = 1.866; *p* = 0.168; Fig. 2*D*; ventral, *F*_(2,18)_ = 0.7764; *p* = 0.4748; Fig. 2*G*; Table S1), indicating that differences in c-Fos labeling were not driven by changes in cell density.

**Figure 3.**
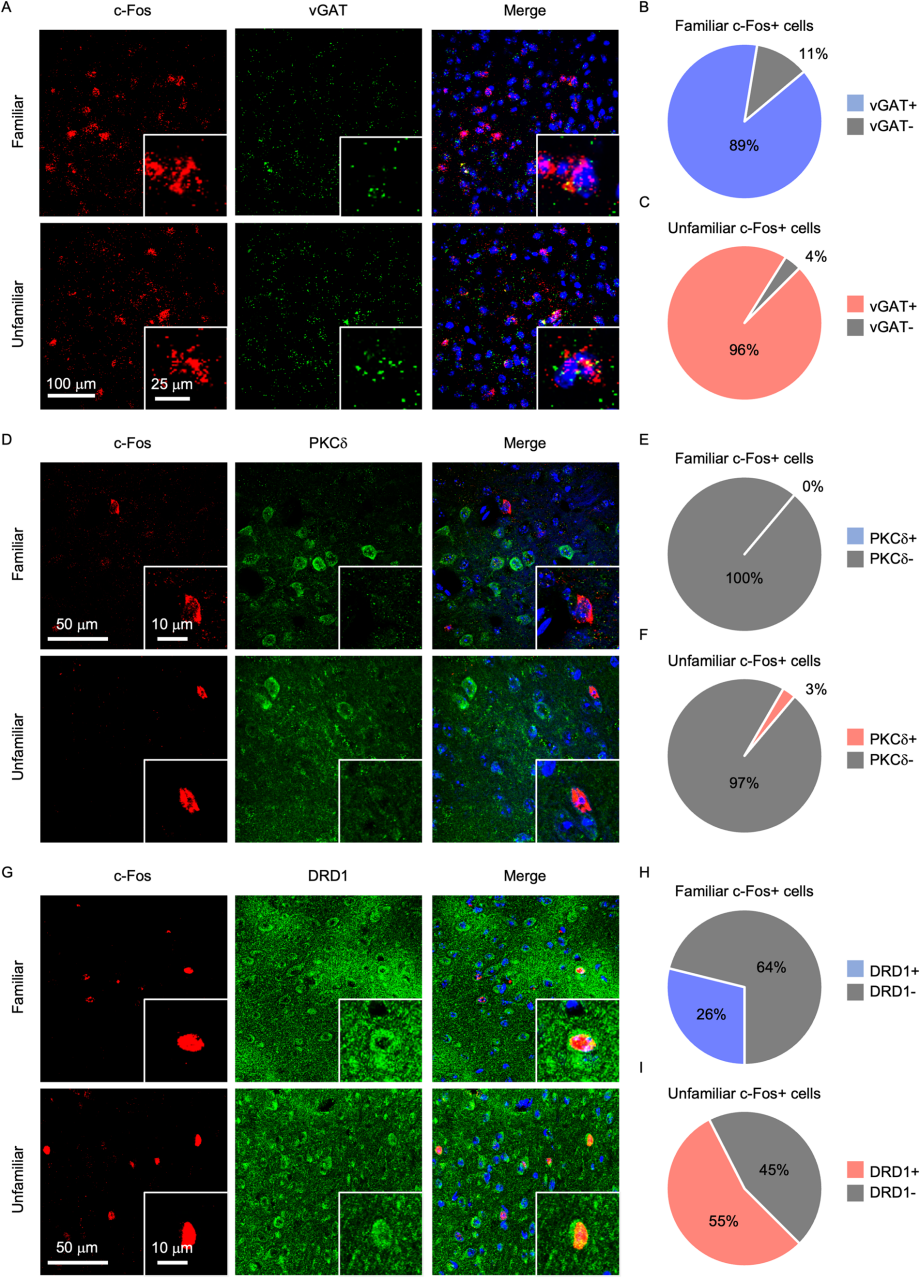
Social interaction predominantly activates inhibitory and dopamine receptor type 1 (DRD1)-expressing neurons in the dorsal BNSTa. ***A***, ***D***, and ***G***, Representative images of tissue labeled with c-Fos, DAPI, and vGAT (***A***), PKCd (***D***), or DRD1 (***G***) after the familiar or unfamiliar free social interaction test. ***B***, ***C***, ***E***, ***F***, ***H***, ***I***, The percentage of c-Fos+ cells that express vGAT (***B***, ***C***), PKCd (***E***, ***F***), and DRD1 (***H***, ***I***) or do not overlap with c-Fos, quantified after the familiar or unfamiliar free social interaction test.

Previous studies have shown that the majority of neurons in the BNSTa are GABAergic (Jennings et al., 2013; Lebow and Chen, 2016; Ortiz-Juza et al., 2021). To determine whether dorsal BNSTa neurons activated by social interaction are inhibitory, we used RNAscope in situ hybridization to visualize c-Fos and vGAT mRNA, with vGAT serving as a canonical marker of inhibitory neurons. RNAscope is a highly sensitive probe-based in situ hybridization method that allows for detection of individual mRNA molecules with minimal background. We focused on the dorsal segment, as no changes in c-Fos were found in the ventral segment relative to control (Fig. 2). We found that the vast majority of c-Fos+ cells in the dorsal BNSTa were vGAT+, regardless of familiarity (familiar, 89% vGAT+; unfamiliar, 96%; Fig. 3*A*–*C*).

The BNSTa contains neuronal populations that express PKCδ and DRD1, which are thought to be largely inhibitory and have been implicated in the regulation of social behavior (Jennings et al., 2013; Kim et al., 2013; Flanigan and Kash, 2020). To determine whether PKCδ+ or DRD1+ neurons in the dorsal BNSTa are recruited during social interaction, we performed immunohistochemical analysis for c-Fos, PKCδ, and DRD1. We observed substantial overlap between c-Fos and DRD1 but little no overlap between c-Fos and PKCδ (familiar PKCδ, 0%; unfamiliar PKCδ, 3%; familiar DRD1, 26%; unfamiliar DRD1, 55%; Fig. 3*D–I*). Notably, nearly three times as many DRD1+ neurons were activated during interaction with an unfamiliar conspecific compared with a familiar.

Together, these findings indicate that social interaction robustly engages GABAergic neurons in the dorsal BNSTa regardless of familiarity, while preferentially recruiting a DRD1+ subpopulation during interaction with unfamiliar conspecifics.

### Dorsal BNSTa neurons are activated during interaction with a conspecific or novel object

To examine changes in neural activity in the BNSTa in real time during familiar and unfamiliar social interaction, we performed calcium imaging using fiber photometry. Fiber photometry is a live imaging technique that captures population-level neuronal activity using fluorescent reporters such as the calcium indicator GCaMP (Dana et al., 2019).

**Figure 4.**
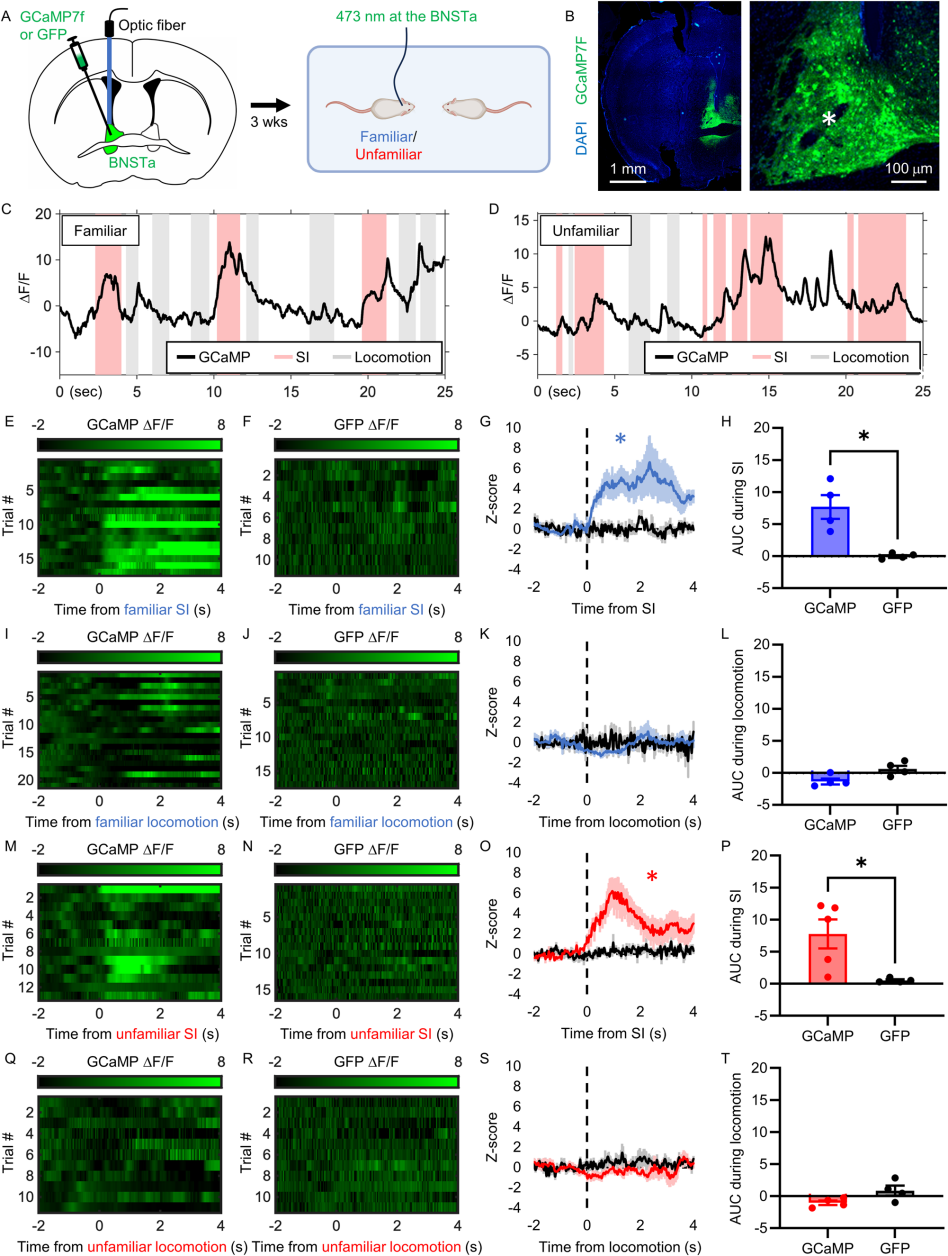
Social interaction, but not locomotion, activates the BNSTa. ***A***, Experimental procedure. Mice were unilaterally injected with GCaMP or GFP control virus into the BNSTa and implanted with an optic fiber into the dorsal BNSTa (*). Three weeks later, dorsal BNSTa activity was measured during the free social interaction test with a familiar or unfamiliar conspecific. ***B***, Representative high- and low-resolution images of GCaMP expression in the BNSTa. ***C***, ***D***, ***E***, ***F***, ***I***, ***J***, ***M***, ***N***, ***Q***, ***R***, Representative graphs and raster plots showing fluorescence changes in the dorsal BNSTa of GCaMP- and GFP-expressing mice interacting with a familiar (***C***, ***E***, ***F***, ***I***, ***J***) or unfamiliar (***D***, ***M***, ***N***, ***Q***, ***R***) conspecific during the free social interaction test, aligned to the start of the interaction (***E***, ***F***, ***M***, ***N***) or general locomotion (***I***, ***J***, ***Q***, ***R***). ***G***, ***K***, ***O***, ***S***, Average *z*-score of fluorescence signal changes in the dorsal BNSTa of mice expressing GCaMP (blue/red trace) or GFP (black trace) interacting with a familiar (***G***, ***K***) or unfamiliar (***O***, ***S***) conspecific before and after the start of the interaction (***G***, ***O***) or general locomotion (***K***, ***S***; *n* = 4 mice per condition). Colored lines indicate group averages and shaded areas indicate SEM. ***H***, ***L***, ***P***, ***T***, Bar graphs comparing the AUC of evoked responses (0–2 s) to social interaction (***H***, ***P***) or general locomotion (***L***, ***T***). Data are mean ± SEM. **p < 0.01; ***p < 0.001.

AAV encoding the fast, ultra-bright calcium indicator GCaMP7f or GFP control was injected into the dorsal BNSTa of 5-to-6-week-old CD1 mice. After 3 weeks, neural activity in the dorsal BNSTa was recorded during the free social interaction test with either a familiar or unfamiliar conspecific (Fig. 4*A*). Representative images of viral expression and fiber placement are shown in Figure 4*B*.

Consistent with our c-Fos findings, interaction with either a familiar or unfamiliar conspecific elicited an increase in the calcium signal in the dorsal BNSTa (familiar, *t*_(6)_ = 2.941; *p* = 0.0259; Fig. 4*C*,*E*,*G*; unfamiliar, *t*_(6)_ = 3.583; *p* = 0.0116; Fig. 4*D*,*M*,*O*; Table S1), indicating neuronal activation. In contrast, no change was observed in GFP-expressing control animals (familiar, *t*_(4)_ = 0.5772; *p* = 0.5947; Fig. 4*F*,*G*; Fig. S1*A*; unfamiliar, *t*_(6)_ = 1.220; *p* = 0.2683; Fig. 4*N*,*O*; Fig. S1*B*; Table S1). Direct comparison of evoked responses revealed significantly greater fluorescence in GCaMP- than GFP-expressing mice during social interaction with both familiar and unfamiliar conspecifics (familiar, *t*_(5)_ = 2.840; *p* = 0.0363; Fig. 4*H*; unfamiliar, *t*_(6)_ = 3.986; *p* = 0.0072; Fig. 4*P*; Table S1).

In contrast to social interaction, general locomotion was not associated with increased calcium activity in the dorsal BNSTa (GCaMP familiar, *t*_(6)_ = 1.351; *p* = 0.2253; Fig. 4*C*,*I*,*K*; GFP familiar, *t*_(4)_ = 0.1859; *p* = 0.8615; Fig. 4*J*,*K*; Fig. S1*A*; GCaMP unfamiliar, *t*_(6)_ = 1.337; *p* = 0.2298; Fig. 4*D*,*Q*,*S*; GFP unfamiliar, *t*_(4)_ = 0.5970; *p* = 0.5826; Fig. 4*R*,*S*; Fig. S1*B*; Table S1). Furthermore, GCaMP and GFP signals did not differ during locomotion in either condition (familiar, *t*_(5)_ = 1.147; *p* = 0.3032; Fig. 4*L*; unfamiliar, *t*_(5)_ = 1.580; *p* = 0.1749; Fig. 4*T*; Table S1).

To assess potential lateralization effects, we performed photometry recordings in a separate cohort of mice injected with GCaMP in the contralateral hemisphere (Fig. S2). Similar increases in calcium activity were observed during interaction with an unfamiliar conspecific (*t*_(4)_ = 3.428; *p* = 0.0266; Fig. S2*C*,*E*,*G*; Table S1). In contrast, locomotion was associated with a decrease in the calcium signal in this hemisphere (*t*_(4)_ = 3.298; *p* = 0.03; Fig. S2*I*,*K*; Table S1), suggesting hemispheric differences in nonsocial processing. No effects were observed in GFP-expressing control mice (unfamiliar interaction, *t*_(6)_ = 0.5285; *p* = 0.6161; Fig. S2*D*,*F*,*G*; locomotion, *t*_(4)_ = 0.5765; *p* = 0.4900; Fig. S2*D*,*J*,*K*; Table S1). Consistent with the primary dataset, interaction with an unfamiliar conspecific—but not locomotion—elicited significantly greater responses in GCaMP- compared with GFP-expressing mice (unfamiliar, *t*_(5)_ = 3.241; *p* = 0.0229; Fig. S2*H*; locomotion, *t*_(4)_ = 1.870; *p* = 0.1348; Fig. S2*L*; Table S1), confirming that dorsal BNSTa responses to unfamiliar social interaction are not hemisphere-specific.

**Figure 5.**
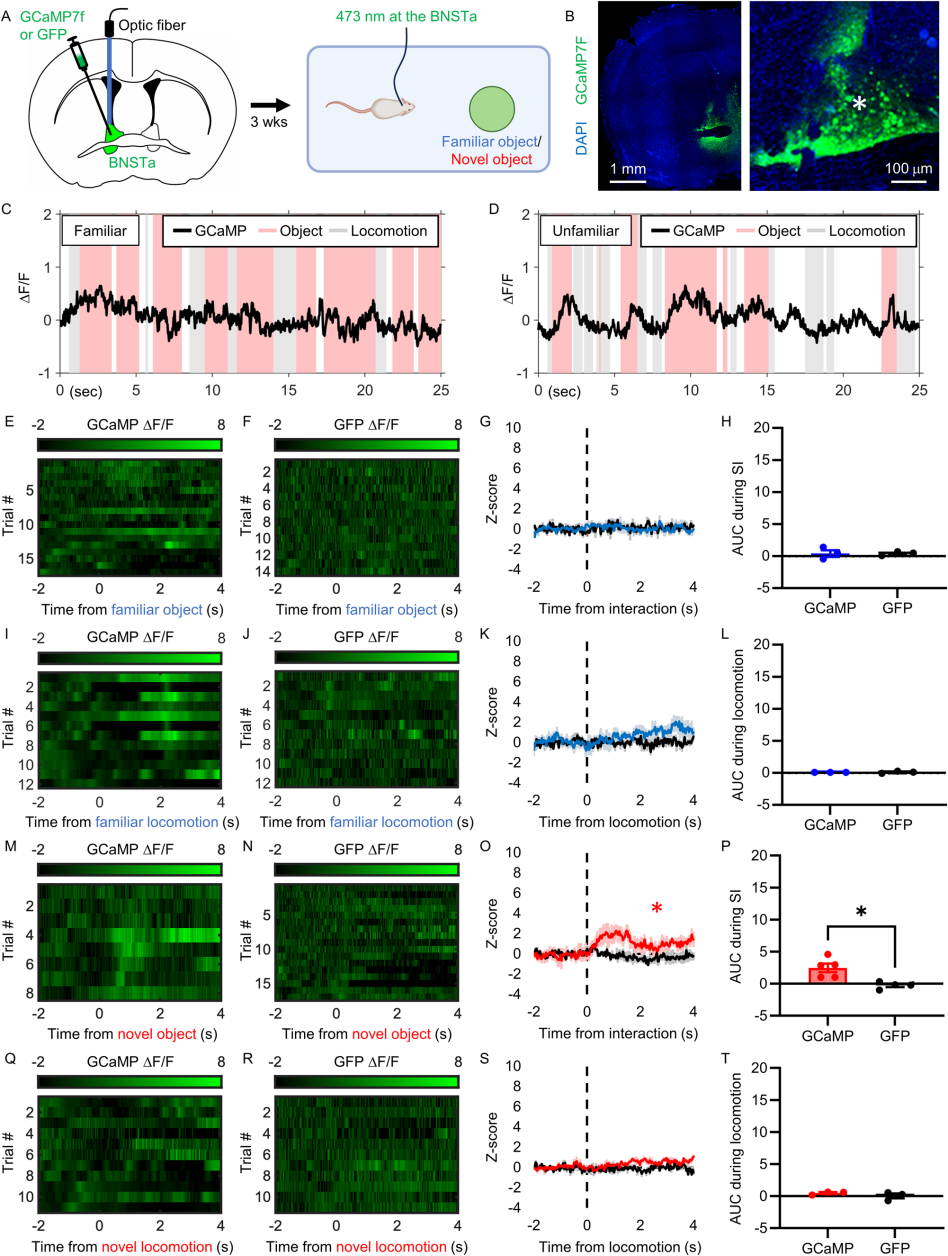
Interaction with novel, but not familiar, objects activates the dorsal BNSTa. ***A***, Experimental procedure. Mice were unilaterally injected with GCaMP or GFP control virus into the BNSTa and implanted with an optic fiber into the dorsal BNSTa (*). Three weeks later, dorsal BNSTa activity was measured during interaction with a familiar or novel object. ***B***, Representative high- and low-resolution images of GCaMP expression in the BNSTa. ***C***, ***D***, ***E***, ***F***, ***I***, ***J***, ***M***, ***N***, ***Q***, ***R***, Representative graphs and raster plots showing fluorescence changes in the dorsal BNSTa of GCaMP- and GFP-expressing mice interacting with a familiar (***C***, ***E***, ***F***, ***I***, ***J***) or novel (***D***, ***M***, ***N***, ***Q***, ***R***) object, aligned to the start of the interaction (***E***, ***F***, ***M***, ***N***) or general locomotion (***I***, ***J***, ***Q***, ***R***). ***G***, ***K***, ***O***, ***S***, Average *z*-score of fluorescence signal changes in the dorsal BNSTa of mice expressing GCaMP (blue/red trace) or GFP (black trace) interacting with familiar (***G***, ***K***) or novel (***O***, ***S***) objects before and after the start of an interaction (***G***, ***O***) or general locomotion (***K***, ***S***). Colored lines indicate group averages, and shaded areas indicate SEM. ***H***, ***L***, ***P***, ***T***, Bar graphs comparing the AUC of evoked responses (0–2 s) during object interaction (***H***, ***P***) or general locomotion (***L***, ***T***). Data are mean ± SEM. **p < 0.01; ***p < 0.001.

Finally, to determine whether dorsal BNSTa activity is selective for social stimuli or reflects a more general novelty signal, we recorded calcium activity during an object interaction test (see Materials and Methods; Fig. 5*A*,*B*). In GCaMP-expressing mice, the dorsal BNSTa responded to interaction with a novel object, but not a familiar object or general locomotion (familiar object, *t*_(4)_ = 0.6435; *p* = 0.5550; Fig. 5*C*,*E*,*G*; locomotion with familiar object, *t*_(4)_ = 1.087; *p* = 0.3560; Fig. 5*C*,*I*,*K*; novel object, *t*_(4)_ = 3.080; *p* = 0.0369; Fig. 5*D*,*M*,*O*; locomotion with novel object, *t*_(4)_ = 0.9125; *p* = 0.4131; Fig. 5*D*,*Q*,*S*; Table S1). No significant effects were observed in GFP-expressing mice under any condition (Table S1). Direct comparison confirmed a significantly greater evoked response to the novel object in GCaMP- compared with GFP-expressing mice (*t*_(4)_ = 3.280; *p* = 0.0305; Fig. 5*P*), with no differences observed for familiar objects or locomotion (Table S1).

Together, these results demonstrate that dorsal BNSTa neurons are engaged during interaction with social and novel stimuli, but not during general locomotion, consistent with the role of the dorsal BNSTa in processing socially relevant and novelty-related information.

### BNSTa inhibition selectively reduces interaction with unfamiliar conspecifics

**Figure 6.**
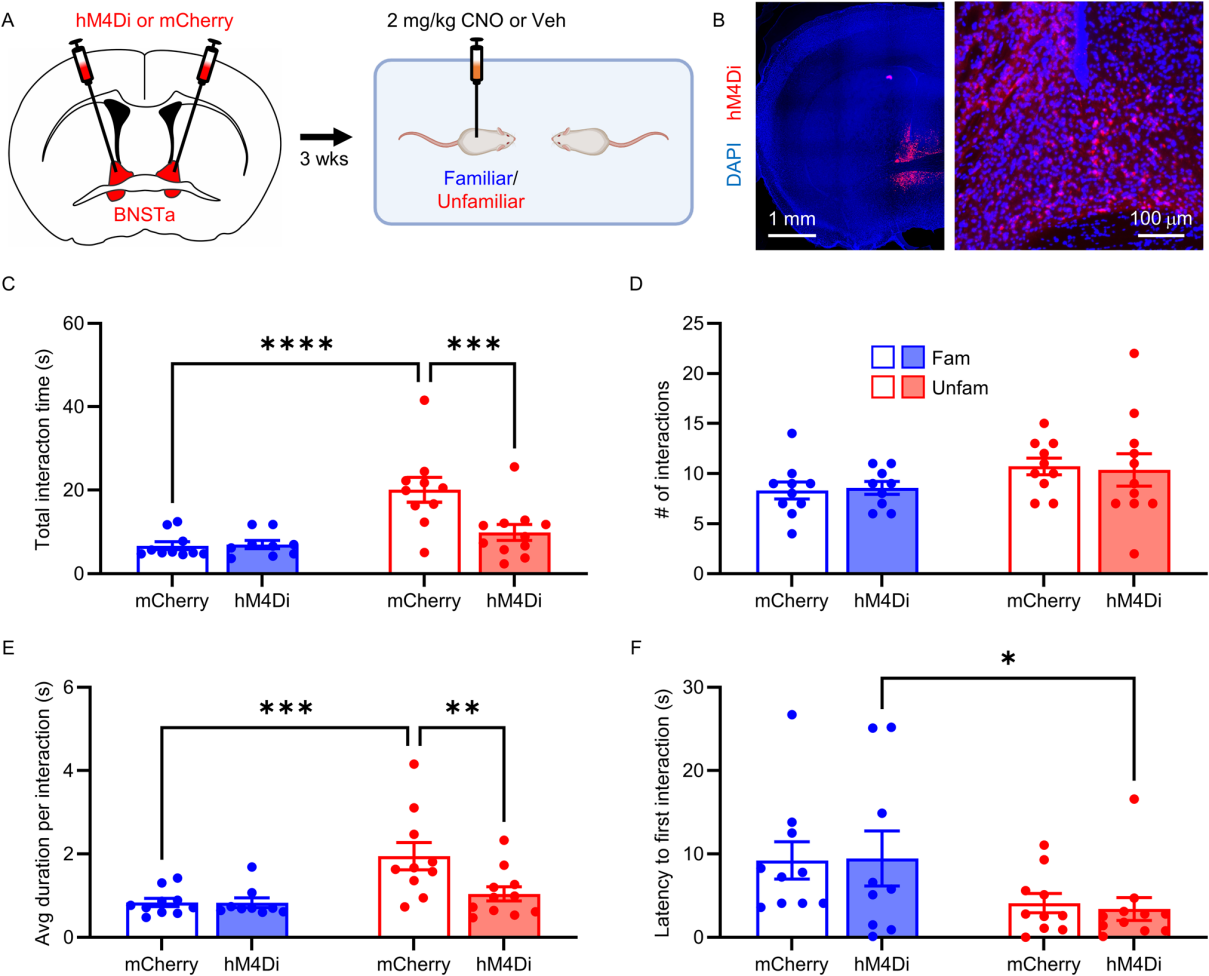
Chemogenetic inhibition of the BNSTa suppresses unfamiliar, but not familiar, social interaction. ***A***, Experimental schedule. Mice were bilaterally injected with hM4Di or mCherry into the BNSTa, followed 3 weeks later by intraperitoneal injections of 2 mg/kg CNO 30 min before the familiar or unfamiliar free social interaction test. ***B***, Representative high- and low-magnification images of hM4Di expression (red) in the BNSTa. ***C–F***, Quantification of social behavior from familiar (blue) or unfamiliar (red) mice expressing hM4Di (closed bar) or mCherry (open bar) and treated with CNO 30 min before the free social interaction test. Mean ± SEM. *p < 0.05; **p < 0.01; ***p < 0.001; ****p < 0.0001.

To examine the causal role of the BNSTa in guiding social interaction, we next used chemogenetics to manipulate BNSTa activity during the free social interaction test. Five-to-six-week-old CD1 mice received injections of either hM4Di or mCherry control virus into the BNSTa. hM4Di is an inhibitory designer receptor exclusively activated by designer drugs receptor that suppresses neuronal activity following ligand binding (Roth, 2016). After 3 weeks of recovery, mice underwent the free social interaction test with either a familiar or unfamiliar conspecific. Thirty minutes prior to testing, subject mice received an intraperitoneal injection of either 2 mg/kg CNO—a dose previously shown to be effective in our studies (Bartsch et al., 2023b; Mojahed et al., 2024; Adjei et al., 2025)—or an equivalent volume of vehicle (Fig. 6*A*). Representative images of viral expression in the BNSTa are shown in Figure 6*B*.

Chemogenetic inhibition of the BNSTa selectively reduced social interaction with unfamiliar, but not familiar, conspecifics. For total social interaction time and average interaction duration, two-way ANOVA revealed a significant interaction between virus and familiarity in CNO-treated animals (total time, *F*_(1,36)_ = 7.33; *p* = 0.010; Fig. 6*C*; duration, *F*_(1,36)_ = 4.88; *p* = 0.034; Fig. 6*E*; Table S1). There was no interaction effect for latency to first interaction; however, a main effect of familiarity was observed (interaction, *F*_(1,36)_ = 0.050; *p* = 0.824; familiarity, *F*_(1,36)_ = 7.140; *p* = 0.011; Fig. 6*F*; Table S1). The number of interactions did not differ across groups (*F*_(1,36)_ = 0.071; *p* = 0.792; Fig. 6*D*; Table S1). Post hoc analyses revealed that CNO treatment in hM4Di-expressing mice reduced total interaction time and duration in unfamiliar pairs to levels comparable to those observed in familiar pairs (total time, *p* = 0.0005; duration, *p* = 0.002; Fig. 6*C*,*E*; Table S1).

Confirming our baseline behavioral findings, vehicle-treated unfamiliar mice exhibited greater social interaction than familiar mice, regardless of viral condition. Two-way ANOVA revealed a main effect of familiarity for total interaction time, number of interactions, and average interaction duration (total time, *F*_(1,36)_ = 21.91; *p* < 0.0001; Fig. S3*A*; number, *F*_(1,36)_ = 7.65; *p* = 0.009; Fig. S3*B*; duration, *F*_(1,36)_ = 11.47; *p* = 0.002; Fig. S3*C*; Table S1). There was no main effect of virus and no virus × familiarity interaction for any measure (Table S1). Latency to first interaction also did not differ across groups or conditions (interaction, *F*_(1,36)_ = 1.99; *p* = 0.166; virus, *F*_(1,36)_ = 0.08; *p* = 0.772; familiarity, *F*_(1,36)_ = 1.80; *p* = 0.188; Fig. S3*D*; Table S1).

**Figure 7.**
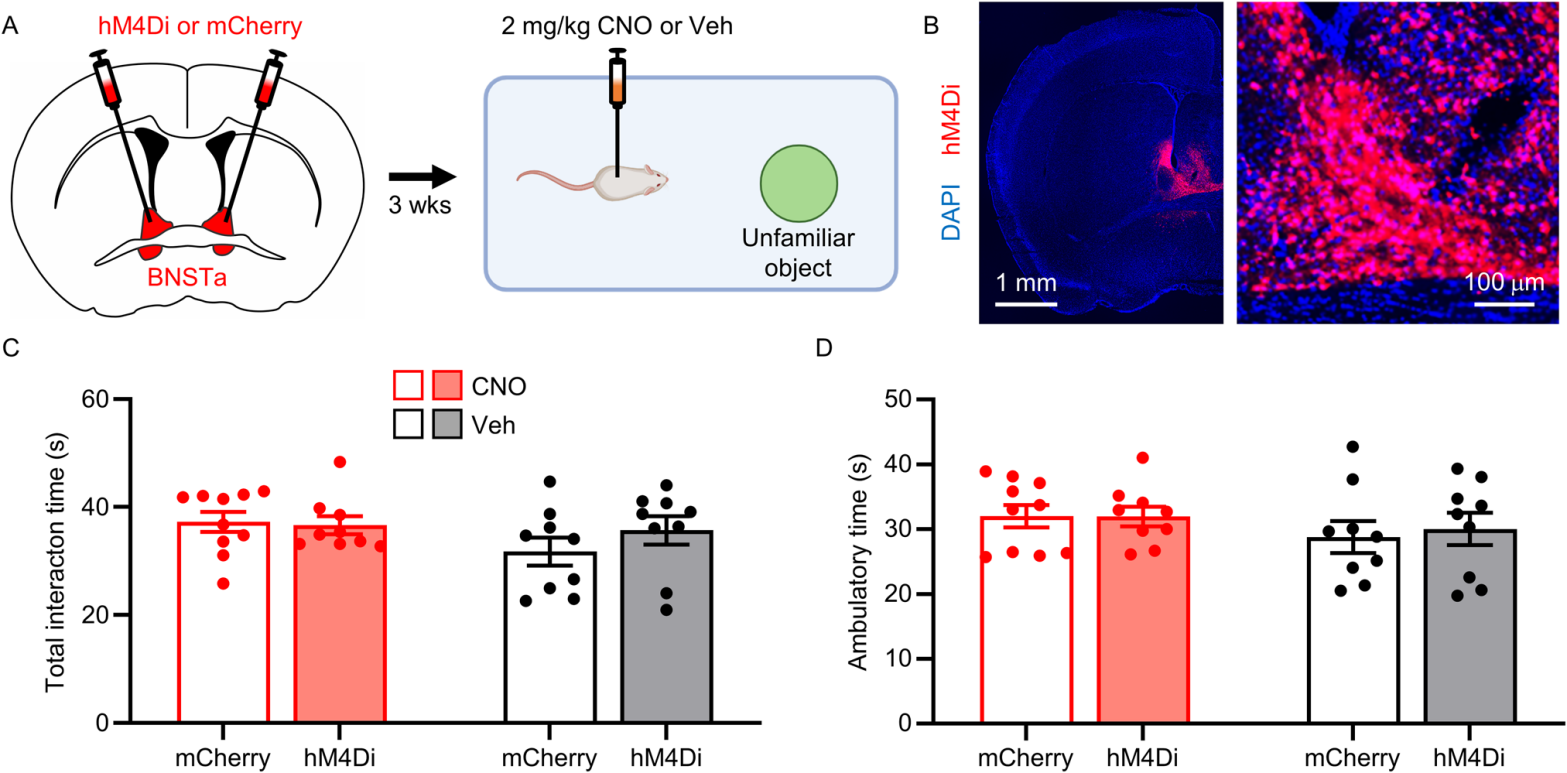
Chemogenetic inhibition of the BNSTa does not affect interaction with a novel object or general locomotion. ***A***, Experimental schedule. Mice were bilaterally injected with hM4Di or mCherry into the BNSTa, followed 3 weeks later by intraperitoneal injections of 2 mg/kg CNO or vehicle 30 min before the object interaction test. ***B***, Representative high- and low-magnification images of hM4Di expression (red) in the BNSTa. ***C–F***, Quantification of total interaction time (***C***) and total ambulatory time (***D***) during the object interaction test. Mean ± SEM.

Finally, because our photometry experiments revealed a significant increase in BNSTa activity during interaction with novel objects, we next asked whether BNSTa inhibition would also suppress novel object interaction, thereby testing whether the BNSTa broadly regulates novelty-driven behavior or is selectively involved in unfamiliar social interaction. Mice expressing hM4Di or mCherry in the BNSTa were treated with CNO or vehicle 30 min prior to the novel object interaction test (Fig. 7*A*,*B*). Chemogenetic inhibition of the BNSTa had no effect on total object interaction time or ambulatory activity (interaction time, *F*_(1,33)_ = 1.056; *p* = 0.311; Fig. 7*C*; ambulatory time, *F*_(1,33)_ = 0.099; *p* = 0.755; Fig. 7*D*; Table S1), indicating that BNSTa activity is not required for novel object exploration or general locomotion.

Together, these findings demonstrate that while BNSTa neurons are engaged during interaction with familiar and unfamiliar conspecifics as well as novel objects, BNSTa activity is selectively required for enhanced social interaction with unfamiliar conspecifics. This establishes a causal and specific role of the BNSTa in promoting novelty-driven social engagement.

## Discussion

Our study is among the first to examine the neural mechanisms underlying differences in free social interaction based on the familiarity of social partners. Most research on social behavior relies almost exclusively on unfamiliar conspecifics, overlooking how familiarity fundamentally shapes social engagement. Here, we incorporated both familiar and unfamiliar conspecifics into a free social interaction test and found that the BNSTa is broadly recruited during social behavior but is selectively required for enhanced interaction with unfamiliar conspecifics. Fiber photometry revealed that neurons in the dorsal BNSTa are activated during both social interaction and novel object exploration, and immunohistochemical analyses suggest that these neurons are predominantly vGAT+ and DRD1+. However, chemogenetic inhibition of the BNSTa reduced social engagement with unfamiliar conspecifics to levels typically observed with familiar cage mates, without affecting interaction with familiar conspecifics, novel objects, or general locomotion. Together, these findings provide causal evidence that the BNSTa is selectively necessary for social novelty preference and point to the role of vGAT+ and DRD1+ neurons in the dorsal BNSTa in supporting this behavior.

### The BNST and social novelty

Social novelty preference is a robust phenomenon across rodent models, with animals consistently spending more time interacting with strangers than familiar cage mates (Kareem and Barnard, 1982; Thor and Holloway, 1982; Kareem, 1983; Moy et al., 2004; Smith et al., 2015). This preference is thought to serve adaptive purposes, enabling evaluation of potential mates, allies, or rivals. Prior work has identified hippocampal CA2, amygdala, and medial prefrontal cortex circuits as central for processing recognition and familiarity (Ferguson et al., 2001; Hitti and Siegelbaum, 2014; Bicks et al., 2015; Okuyama et al., 2016; Meira et al., 2018; Tan et al., 2019). Our findings extend this framework by showing that the BNSTa, a critical node in the social behavior network, does not encode recognition itself but may act as a site where familiarity signals are translated into behavioral output. This highlights the value of the free social interaction test, which, unlike the three-chamber assay that primarily measures recognition memory, captures how familiarity shapes the structure of ongoing social behavior (Kondrakiewicz et al., 2019; Jabarin et al., 2022; Szabó et al., 2024).

Although prior studies have implicated BNST circuits in social behavior, none have directly assessed how BNST activity differs when interacting with familiar versus unfamiliar conspecifics. Instead, most evidence has come from related lines of work that indirectly suggest the role of the BNST in novelty- or recognition-related processes. For example, chemogenetic modulation of the BNST can normalize disrupted novelty preference following early-life stress, pointing to BNST involvement in novelty-related social processes (Emmons et al., 2021). Additional evidence comes from the gut–brain axis, where microbial metabolites disrupt social recognition through activation of BNST CaMKIIα+ neurons, and inhibition of these neurons rescues the deficit (Liou et al., 2023). Neuropeptide signaling provides another window into familiarity-sensitive mechanisms: vasopressinergic BNST projections to the lateral septum increase time spent near an unfamiliar conspecific, and ablating BNST vasopressin neurons or blocking oxytocin signaling disrupts social recognition memory in males (Dumais et al., 2016; Whylings et al., 2020; Rigney et al., 2024). Taken together, these findings converge on the idea that the BNST is tuned to social novelty and recognition, shaping how animals engage with unfamiliar individuals. Our results build directly on this body of work by demonstrating that BNSTa activity is broadly engaged during social interaction but selectively required for social novelty preference, establishing the causal role of the BNSTa in familiarity-dependent social behavior.

We found that over half (55%) of dorsal BNSTa cells expressing c-Fos following interaction with an unfamiliar conspecific were DRD1+, compared with nearly one-quarter (26%) following familiar social interaction. This enrichment suggests that dopamine-sensitive dorsal BNSTa neurons are preferentially recruited during social novelty. The BNSTa receives dopaminergic input from the ventral tegmental area and dorsal raphe nuclei (Meloni et al., 2006; Matthews et al., 2016; C. R. Lee et al., 2025), and prior work has implicated BNST dopamine signaling in sociability and exploratory behavior (Matthews et al., 2016; C. R. Lee et al., 2025). Whether dopamine input to the dorsal BNSTa plays a role in social novelty seeking remains to be addressed. Future studies leveraging cell-type–specific approaches and direct measurements of dopamine signaling will be important for resolving this question.

### Valence surveillance and mechanistic interpretations

The BNST has traditionally been studied in the context of sustained fear and anxiety, in contrast to the amygdala's role in rapid, phasic responses (Walker et al., 2003; Walker and Davis, 2008; Davis et al., 2010). More recent work shows that BNST circuits also regulate motivated behaviors such as feeding, drug seeking, aggression, and social interaction (Avery et al., 2016; Lebow and Chen, 2016; Flanigan and Kash, 2020; Ortiz-Juza et al., 2021). This diversity has led to the proposal that the BNST functions as a “valence surveillance” system, integrating internal state with external cues to bias behavior under uncertain conditions (Lebow and Chen, 2016; Flanigan and Kash, 2020). According to this framework, the BNST does not encode recognition directly but instead evaluates the salience and potential valence of stimuli, shaping behavioral outcomes accordingly.

Our findings are consistent with this interpretation. BNSTa neurons were engaged during both familiar and unfamiliar encounters, consistent with salience detection, but chemogenetic inhibition revealed a selective requirement for enhanced interaction with strangers. This suggests that BNSTa activity amplifies the incentive value—or positive valence—of novelty, engaging outputs that bias social behavior toward unfamiliar conspecifics. Consistent with this idea, our activity-mapping data indicate that social interaction preferentially recruits the dorsal BNSTa—a subdivision linked to approach-related behaviors—while sparing ventral BNST regions more closely associated with social threat processing (Flanigan and Kash, 2020; Jacobs et al., 2023; Wright et al., 2023).

Because the vast majority of BNSTa neurons recruited during social interaction were vGAT+, the above effects are likely mediated by inhibitory projections, potentially via disinhibition of downstream targets that promote affiliative behavior. One candidate is the lateral hypothalamus, where a GABAergic BNST projection has been shown to increase general approach behavior (Giardino et al., 2018). Another possibility is the ventral tegmental area, as BNST GABAergic inputs to the VTA can regulate dopamine release (Kudo et al., 2012), raising the possibility that novelty preference is reinforced through dopaminergic mechanisms. Similarly, projections to the lateral septum may contribute by modulating social investigation (Rigney et al., 2024). Determining the relative contribution of these pathways will be critical for clarifying how BNSTa activity promotes approach toward unfamiliar conspecifics.

Importantly, BNST contributions to behavior are highly state dependent and strongly modulated by stress and anxiety, with BNST activity scaling with contextual threat, uncertainty, and anxious temperament in humans and nonhuman primates (Kalin et al., 2005; Fox et al., 2015; Shackman and Fox, 2016). Consistent with this view, BNST responses have been shown to shift toward threat-related signaling under stressful social conditions (Wright et al., 2023). Within this framework, the novelty-promoting role of the BNSTa observed here likely reflects a low-stress baseline condition, whereas under heightened stress or threat, unfamiliar social stimuli may instead recruit BNSTa circuits that bias behavior toward vigilance or avoidance.

Together, these results suggest that under baseline conditions, the BNSTa serves as an integration hub, combining salience and valence signals with projection-specific outputs to bias social behavior toward unfamiliar partners.

### Limitations and future directions

While our findings establish the clear role of the BNSTa in regulating familiarity-dependent social interaction, several points warrant further investigation. First, the free interaction test we used captures highly ethological dynamics but is inherently reciprocal, making it difficult to disentangle the subject's social behavior from that of the conspecific. Second, our study was conducted in males, and although this approach provided a consistent baseline, the BNSTa is strongly sexually dimorphic (Allen and Gorski, 1990; Whylings et al., 2020), making the extension of this work to females an important next step. Third, while the BNSTa comprises medial and lateral subdivisions with overlapping connectivity and function across species (Fox et al., 2015), our recordings and manipulations span both components, limiting interpretability across this axis. Finally, although BNSTa activity was observed during both familiar and unfamiliar social interactions, these responses are likely distributed across distinct neuronal populations. Consistent with this idea, we find that DRD1+ neurons in the dorsal BNSTa are preferentially recruited during interaction with unfamiliar conspecifics, suggesting cell-type–specific contributions to familiarity-dependent social behavior that will be important to resolve in future studies.

Future studies should address these questions by identifying the downstream pathways through which BNSTa inhibitory neurons influence social approach. Including female cohorts and manipulating sex hormones will also be important to capture sex-dependent mechanisms, especially given evidence that BNST neuropeptide circuits regulate male-specific social recognition memory (Dumais et al., 2016; Whylings et al., 2020; Rigney et al., 2024). Combining these circuit- and state-level approaches with high-resolution tools will provide deeper insight into how the BNSTa integrates familiarity, motivation, and salience to guide social decision-making.

### Clinical implications and conclusion

These findings also have translational relevance. Dysregulated BNST activity has been implicated in neuropsychiatric conditions marked by social deficits, including anxiety disorders, schizophrenia, and ASD (Pierce et al., 2004; Lebow et al., 2012; Clauss et al., 2019; Figel et al., 2019; Feola et al., 2021). Importantly, these disorders feature impairments in novelty processing—whether excessive avoidance of unfamiliar social partners, as in social anxiety, or reduced discrimination between familiar and novel individuals, as in ASD. Our results demonstrate that BNSTa activity is selectively required for approach toward unfamiliar conspecifics, pointing to a mechanism by which BNSTa dysregulation could distort novelty-driven social behavior. Considering the strong sexual dimorphism of the BNST, such dysfunction may also contribute to sex differences in vulnerability to these disorders. Together, our findings highlight the BNSTa as a key node linking social recognition to behavioral output, shaping how animals engage with unfamiliar conspecifics and navigate the uncertainty of novel social encounters.
